# Retraction: *Eafs* Control Erythroid Cell Fate by Regulating *c*-*myb* Expression through Wnt Signaling

**DOI:** 10.1371/annotation/6adbe6d0-19e7-4b1c-a300-e3535c35ce71

**Published:** 2013-08-12

**Authors:** Xufa Ma, Jing-Xia Liu

The editors and the authors retract this publication following the authors' request.

Following the publication of this article, the following concerns were raised in relation to this work:

- The original data for the study as well as Figures A1-A3 and Figure 2 originate from the laboratory of Wuhan Xiao Institute of Hydrobiology, Chinese Academy of Sciences, and were in part conducted by a researcher in that laboratory who was not listed on the authorship byline of the paper. The data in these figures were published without the permission or knowledge of Dr Xiao and of the researcher who conducted the research underlying those figures.

- In addition to the funding source stated on the published article, which corresponds to a grant assigned to Dr. Jing-Xia Liu and held by Ministry of Science and Technology of the People’s Republic of China and Chinese Academy of Science, grants assigned to Wuhan Xiao also supported this work: "973" grant 2010CB126306, NSFC grant 91019008, 31071212 and National Transgene Project 2009ZX08010-021B. 

The academic committee at Institute of Hydrobiology, Chinese Academy of Sciences has carried out an investigation into the work reported in the article and has agreed with the authors’ request of retraction of the publication.

The authors would like to apologize to the readers and Dr Xiao.

